# Gamma motor neurons express distinct genetic markers at birth and require muscle spindle-derived GDNF for postnatal survival

**DOI:** 10.1186/1749-8104-4-42

**Published:** 2009-12-02

**Authors:** Neil A Shneider, Meghan N Brown, Courtney A Smith, James Pickel, Francisco J Alvarez

**Affiliations:** 1 Department of Neurology, Center for Motor Neuron Biology and Disease, Columbia University, New York, New York 10032, USA; 2 Section on Developmental Neurobiology, National Institute of Neurological Disorders and Stroke, National Institutes of Health, Bethesda, Maryland 20892, USA; 3 Department of Neurosciences, Cell Biology, and Physiology, Wright State University, Dayton, Ohio 45435, USA; 4 National Institute of Mental Health Transgenic Core, National Institutes of Health, Bethesda, Maryland 20892, USA

## Abstract

**Background:**

Gamma motor neurons (γ-MNs) selectively innervate muscle spindle intrafusal fibers and regulate their sensitivity to stretch. They constitute a distinct subpopulation that differs in morphology, physiology and connectivity from α-MNs, which innervate extrafusal muscle fibers and exert force. The mechanisms that control the differentiation of functionally distinct fusimotor neurons are unknown. Progress on this question has been limited by the absence of molecular markers to specifically distinguish and manipulate γ-MNs. Recently, it was reported that early embryonic γ-MN precursors are dependent on GDNF. Using this knowledge we characterized genetic strategies to label developing γ-MNs based on GDNF receptor expression, showed their strict dependence for survival on muscle spindle-derived GDNF and generated an animal model in which γ-MNs are selectively lost.

**Results:**

In mice heterozygous for both the *Hb9::GFP *transgene and a tau-lacZ-labeled (*TLZ*) allele of the GDNF receptor Gfrα1, we demonstrated that small motor neurons with high Gfrα1-TLZ expression and lacking Hb9::GFP display structural and synaptic features of γ-MNs and are selectively lost in mutants lacking target muscle spindles. Loss of muscle spindles also results in the downregulation of Gfrα1 expression in some large diameter MNs, suggesting that spindle-derived factors may also influence populations of α-MNs with β-skeletofusimotor collaterals. These molecular markers can be used to identify γ-MNs from birth to the adult and to distinguish γ- from β-motor axons in the periphery. We also found that postnatal γ-MNs are also distinguished by low expression of the neuronal nuclear protein (NeuN). With these markers of γ-MN identity, we show after conditional elimination of GDNF from muscle spindles that the survival of γ-MNs is selectively dependent on spindle-derived GDNF during the first 2 weeks of postnatal development.

**Conclusion:**

Neonatal γ-MNs display a unique molecular profile characterized by the differential expression of a series of markers - Gfrα1, Hb9::GFP and NeuN - and the selective dependence on muscle spindle-derived GDNF. Deletion of GDNF expression from muscle spindles results in the selective elimination of γ-MNs with preservation of the spindle and its sensory innervation. This provides a mouse model with which to explore the specific role of γ-fusimotor activity in motor behaviors.

## Background

Muscle spindles provide proprioceptive information required for motor control. Unlike other mechanoreceptors, the sensitivity of muscle spindles is actively regulated by a specialized fusimotor system. This allows for continuous control of the mechanical sensitivity of spindles over the wide range of lengths and velocities that occur during normal motor behaviors [1]. Fusimotor axons originate either from gamma motor neurons (γ-MNs), which only innervate intrafusal fibers of the muscle spindle, or from alpha motor neurons (α-MNs), which innervate extrafusal muscle and also send a β-skeletofusimotor collateral axon to innervate the muscle spindle [2-4]. Phylogenetically, γ-MNs are best developed in mammals, whereas lower vertebrates (for example, amphibians) use a β-skeletofusimotor system alone to control the sensitivity of their muscle spindles. The advantages of a γ-fusimotor system to control spindle sensitivity independently of force-generating extrafusal muscle fibers are not fully understood, nor are the mechanisms that generate the distinct γ- and α-MN subtypes in mammals.

Most motor pools contain both α- and γ-MNs, which derive from common progenitors and then differentiate to form specific cell types that differ in morphology, physiology and connectivity (for reviews, see [1,5]). Investigation of the mechanisms that control γ- from α-MN differentiation has been limited by the lack of available molecular markers to distinguish these functionally distinct subpopulations during development, as molecular differences between postnatal α- and γ-MNs have only recently been demonstrated [6]. Without such selective markers, γ-MN identity has been based routinely on cell size or physiological differences in conduction velocity. However, early in postnatal development when differences in MN cell diameter are less apparent [7,8] it is not possible to distinguish α- from γ-MNs by size alone. This is also true in adult motor pools with intermediate cell diameters [9,10]. In addition, conduction velocity does not mature until myelination is complete late in development. The lack of criteria for γ-MN identification during development has thus hindered the study of the differentiation of the fusimotor system and the specific roles played by γ-MNs in motor control.

Recent work has shown that survival of γ-MN precursors during embryonic development is selectively dependent on glial cell line-derived neurotrophic factor (GDNF) [11,12]. Absence of GDNF signaling before the induction of muscle spindles in early embryos resulted in the loss of γ-MNs. These observations raise questions about whether muscle spindles and spindle-derived GDNF are important for the differentiation or survival of γ-MNs during late embryonic and postnatal development when specific characteristics of γ-MNs, such as intrafusal innervation, smaller cell body size and differences in axon myelination, emerge. Is the muscle spindle a required source of GDNF? Does spindle-derived GDNF function in trophic support of γ-MNs, or some other aspect of fusimotor differentiation and function? Does the loss of spindle GDNF have consequences for motor behaviors?

To address these issues, we investigated the expression pattern of the GDNF receptor Gfrα1 and that of several other markers in postnatal MNs and defined molecular and genetic criteria that can be used to identify postnatal γ-MN somata independent of size and to distinguish γ- from β-fusimotor axons in the periphery. Furthermore, genetic elimination of spindle-derived GDNF using a novel conditional allele of the *GDNF *gene (*GDNF*^*FLOX*^) demonstrates that the selective dependence of γ-MN survival on GDNF continues after birth and that muscle spindles are a critical source of this factor in the postnatal period. The conditional elimination of GDNF expression from muscle spindles in the mouse results in selective γ-MN loss with no obvious effect on other spindle components or α-MNs, and so provides a model to investigate the specific role of γ-fusimotor activity in motor behaviors.

## Materials and methods

### Mouse genetics: generation of the *GDNF*^*FLOX *^allele

Mouse GDNF genomic clones from a 129sv/J genomic library were kindly provided by Jose Pichel [13]. The targeting vector for the *GDNF*^*FLOX *^allele was constructed from an approximately 8 kb *Sph*I/*Nco*I fragment containing the GDNF coding sequence of exon 3. *loxP *sites were placed in the intronic sequence just upstream of exon 3 and in the 3' untranslated region of the *GDNF *gene. A neomycin-resistance expression cassette flanked by FRT sites was inserted upstream of the 5' *loxP *site. The linearized targeting construct was electroporated into W9.5 embryonic stem cells, selected with G418 and screened for homologous recombinants by Southern analysis (*Eco*RV digest) using as probe a 2-kb fragment downstream of the 3' end of the targeting construct. The frequency of recombination with this construct was low (<1%). Recombinant clones were injected into C57BL/6J blastocysts to generate chimeric founders. After germline transfer of the *GDNF*^*FLOX*^*+NEO *allele was confirmed, the neomycin cassette was excised by crossing F1 animals to ACTB-FLPe mice [14] to generate the *GDNF*^*FLOX *^allele. The *GDNF*^*FLOX *^allele was maintained on a predominantly C57BL/6J strain background.

Other mouse lines used in this study were previously characterized and generously shared, and include Gfrα1-TLZ [15], *Hlxb9-GFP1Tmj *(*Hb9::GFP*) [16], *Egr3*^*NULL *^[17], *ErbB2*^*FLOX *^[18], *ErbB2*^*NULL *^[19], *myf5-CRE *[20], and *GDNF*^*LacZ *^[21]. All animal studies were performed under an approved IACUC animal protocol according to the institutional guidelines of the National Institute of Neurological Disorders and Stroke, and the College of Physicians and Surgeons at Columbia University.

### *In vivo *retrograde labeling of motor pools from identified muscle

At postnatal day 18, animals were deeply anesthetized by halothane induction, and individual muscles were surgically exposed and pressure injected using a glass micropipette with 1 to 2 μl of a 2.5% solution of Fast Blue diluted in 0.01 M phosphate buffered saline (PBS; EMS-Polyloy, Groβ-Umstadt, Germany). After recovery from surgery, animals were held for 48 to 72 hours, and then transcardially perfused with 4% paraformaldehyde diluted in 0.01 M PBS. Isolated spinal cords were immersion-fixed overnight in the same fixative solution and washed in PBS before processing for immunocytochemical analysis.

### Immunohistochemistry

Spinal cords were dissected from postnatal day (P)5, P20 or P60 animals that were transcardially perfused with PBS followed by 4% paraformaldehyde and postfixed overnight, as above. For sectioning, the spinal cords were embedded in warm 5% agar and serial transverse sections (75 μm thick) cut in a vibratome and processed free-floating. The sections were blocked with 10% normal donkey serum diluted in PBS with 0.1% Triton X-100 (pH 7.4) and incubated overnight at room temperature in primary antisera diluted in PBS with 0.1% Triton X-100. The following day, immunoreactive sites were revealed with different species-specific goat or donkey secondary antibodies, depending on the experiment, and coupled to Alexa 488, 555, 647 (dilutions 1:250 to 1:1,000; Invitrogen, Carlsab, CA, USA), Cy3 or Cy5 (dilution 1:50 to 1:100; Jackson Immunoresearch Labs, West Grove, PA, USA). All fluorescent sections were mounted in anti-fading solution Glycerol:PBS (3:7) or Vectashield (Vector Labs, Burlingame, CA, USA).

For analysis of neonatal spinal cords and muscle spindles, tissues were fixed overnight in 4% paraformaldehyde, washed in PBS, cryoprotected in 30% sucrose in 0.1 M phosphate buffer (PB) and then frozen in OCT (ProSciTech, Queensland, Australia). Cryostat sections were cut at a thickness of 14 to 20 μm and placed on glass slides for immunostaining using the same conditions as above.

Primary antibodies used in this study were specific for vesicular glutamate transporter 1 (VGluT1; dilution 1:10,000; guinea pig polyclonal, gift of Julia Kaltschmidt and Tom Jessell), choline acetyl transferase (ChAT; dilution 1:250; goat polyclonal, AB144, Millipore, Temecula, CA, USA), vesicular acetylcholine transporter (VAChT; dilution 1:500; guinea pig polyclonal, AB1588, Millipore); β-galactosidase (dilution 1:1,000; chick polyclonal, ab9361, AbCam, Cambridge, MA). Hb9::GFP fluorescence in MNs was detected without the use of immunocytochemical amplification, but in the periphery, green fluorescent protein (GFP) in motor axons was visualized with a sheep anti-GFP polyclonal antibody (1:1,000; 4745-1051, Biogenesis, Brentwood, NY, USA). Muscle spindles and motor axons in muscle sections were labeled with antibodies against the peripheral axon marker protein gene product 9.5 (PGP9.5; 1:500; rabbit polyclonal antibody, 7863-0504 AbD Serotec, Raleigh, NC, USA) and acetylcholine receptor clusters at intrafusal and extrafusal neuromuscular junctions were labeled with Cy5-bungarotoxin (1:1,000; Molecular Probes, Invitrogen).

### Motor neuron counts and size distribution histograms

At each postnatal age, counts of MNs were performed on the lateral motor column of lumbar spinal segments L4 through L5 from z-series of confocal optical sections obtained at a magnification of 20× (0.9× optical zoom; z-step of 2.5 μm). Motor neurons labeled with a combination of ChAT, β-galactosidase and GFP were counted and measured using Neurolucida (Microbrightfield Bioscience, Williston, VT, USA). All ChAT+ MNs imaged in each stack were outlined in the confocal plane where each exhibited the maximum cell body cross-section and classified according to their differential expression of Gfrα1-TLZ and Hb9::GFP. Distribution histograms were constructed for each animal by grouping cell body cross-sectional areas in 50 μm^2 ^bins. In each animal distribution, histograms represent pooled data from six ventral horns. Depending on age, genotype and section thickness, approximately 40 to 80 MNs were counted per ventral horn. A minimum of three distribution histograms from three different animals of similar age/genotype were averaged in each experiment (exact numbers provided in the Results section). Average histograms were fit to either single or dual Gaussian distributions using Clampfit (version 9.0; Axon Instruments, Union City, CA, USA). From the fitted distributions we estimated the average cross-sectional area and standard deviation (SD) of the small and large size MN populations. From the raw histogram data we obtained relative percentages for each population according to cell size or phenotype. In the histograms, error bars always represent ± standard error of the mean (SEM). Depletions in certain genotypes were calculated against all ChAT+ MNs or the number of cells identified by a particular set of markers or cells below a certain threshold cutoff size. Cutoff sizes for the small population were estimated as the average (μ) + 2 SD (σ) of the fitted small population distribution in control animals of similar age.

### VGluT1 and VAChT contact counts

Quantitative analysis of VGluT1 and VAChT immunoreactive (VGluT1+ and VAChT+, respectively) synaptic densities on MNs at postnatal day 20 were performed on z-series of optical confocal sections obtained at high magnification (63×, 1.4 NA, 1.0× optical zoom, z-step, 1 μm) throughout the entire cell body and proximal dendrites of randomly selected large diameter, Hb9::GFP+/Gfrα1-TLZ-/ChAT+ and small Hb9::GFP-/Gfrα1-TLZ+/ChAT+ MNs. VGluT1+ and VAChT+ contacts were counted over the surface of each MN. Contact densities on cell somata were estimated by measuring the diameter of each MN cell body in all three planes and calculating the surface area of each cell - modeled as an ellipsoid - as previously described [22]. Contact numbers on dendrites were normalized against the length of the dendritic segments measured in two-dimensional projections of the three-dimensional confocal image stacks. Average densities on Hb9::GFP+/Gfrα1-TLZ-/ChAT+ MNs were compared to small Hb9::GFP-/Gfrα1-TLZ+/ChAT+ MNs using *t*-tests.

### Neurolucida reconstruction of α and γ motor neurons

To analyze MN morphology and dendritic structure, confocal images obtained from P20 *Gfrα1-TLZ*/*Hb9::GFP *compound heterozygous animals were used to reconstruct individual Hb9::GFP+/Gfrα1-TLZ-/ChAT+ and Hb9::GFP-/Gfrα1-TLZ+/ChAT+ MNs using Neurolucida software (MicroBrightfield, Williston, VT, USA). The MN cell body was traced in a middle cross-sectional optical section. Dendrite origins were located at the points at which the membrane changed from convex out to concave out. Each dendrite was manually traced by making discrete measurements along their paths and manually entering each branching point. Dendrite thickness was first entered at dendrite origins (by adjusting cursor size) and then readjusted at each measurement point. Reconstructed neurons were analyzed with Neuroexplorer software (version 8.0, MicroBrightfield) to obtain information on the number of primary or higher order dendrites, their average thickness, branching patterns, total dendritic tree length, total dendrite surface and dendritic lengths and surfaces of individual dendrites or dendritic segments at different Sholl distances. Sholl analysis calculated the amount of surface membrane in dendritic segments at different distances from the cell body. For this purpose a set of nested concentric spheres was centered at the cell body with the spheres separated by 50 μm, creating a series of shells of increasing distance from the cell body. The total surface area of all dendrite segments contained within each shell was added to obtain an estimate of the distribution of dendritic surface membrane at increasing distances from the cell body. Fine caliber distal dendrites were better resolved with the Gfrα1-TLZ marker compared to Hb9::GFP-labeled dendrites. As a result, longer dendritic segments were analyzed in Gfrα1-TLZ+ MNs. Therefore, the most meaningful comparisons focused on the more proximal dendritic tree that was equally sampled in both MN cell types.

### *In situ *hybridization

*In situ *hybridization analysis was performed with digoxigenin-labeled cRNA probes [23] specific for Egr3 [24] and GDNF. The sequence of the entire murine GDNF coding region was amplified by RT-PCR of embryonic day 10.5 (E10.5) mouse RNA, cloned into pBluescript SK vector (Stratagene, Inc., La Jolla, CA, USA) and sequenced. GDNF riboprobes were prepared by *in vitro *transcription of the GDNF cDNA.

### Semithin section analysis of muscle spindles

One P18 *GDNF*^*FLOX*/*FLOX *^*Egr3*^*CRE*/*CRE *^double homozygote and one P18 *GDNF*^*FLOX*/+ ^*Egr3*^+/+ ^control animal were transcardially perfused with 4% paraformaldehyde and 4% glutaraldehyde diluted in 0.1 M PB. The tibialis anterior muscle (studied here) and several others were dissected, washed, postfixed with 2% OsO_4 _in 0.1 M PB and embedded in Spurr resin. Serial semithin sections were obtained transversally through the blocks, contrasted with 1% toluidine blue/borax and the equatorial and polar regions of muscle spindles imaged with bright field light microscopy. Images were digitally recorded with a 60× or 100× objective and using a RT-Spot Camera (Diagnostic Instruments, Sterling Heights, MI, USA).

## Results

### Gfrα1 expression is restricted to subpopulations of motor neurons in the lumbar spinal cord

Previous studies have demonstrated that Gfrα1 is expressed in a subset of MNs [25-28] and that GDNF/Gfrα1 signaling is required for the survival of spindle-innervating MNs [11,12]. These findings suggest that Gfrα1 may be a marker for γ-MNs. In this study, we characterized the subpopulation of Gfrα1+ spinal MNs in mice heterozygous for a null allele of Gfrα1 marked by the expression of tau-lacZ (Gfrα1-TLZ) [15] and the *Hb9-GFP1Tmj *transgene (Hb9::GFP). In Hb9::GFP animals, the murine *HB9 *promoter drives expression of enhanced GFP in MNs and a subpopulation of ventral interneurons [16,29,30].

**Figure 1:**
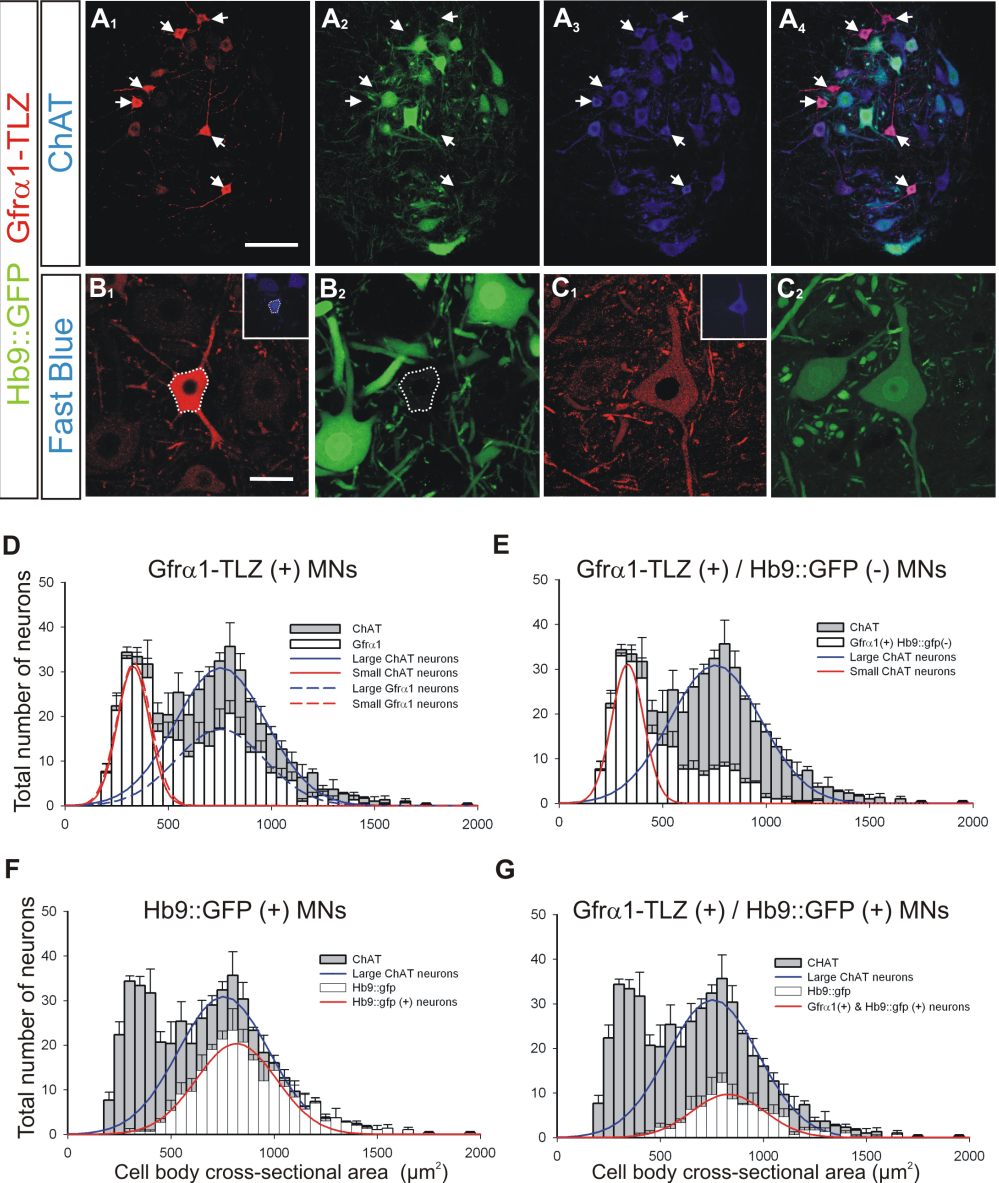
**Small size motor neurons in postnatal day 20 mouse spinal cords are strongly Gfrα1-TLZ positive and Hb9::GFP negative**. **(A) **Confocal images of lumbar lamina IX showing Gfrα1-Tau-lacZ (TLZ) immunoreactivity (A_1_, red, cy3) and Hb9::GFP expression (A_2_, green) in ChAT+ MNs (A_3_, blue, Cy5); merged images in A_4_. Gfrα1-TLZ strongly positive MNs are small and Hb9::GFP negative (arrows in A_1-4_). **(B) **Small, strongly Gfrα1-TLZ positive MN (B_1_) retrogradely labeled from tibialis anterior muscle (Fast Blue, inset). This MN lacks Hb9::GFP (B_2_) (cell body location outlined). **(C) **Medium size MN with weak Gfrα1-TLZ immunoreactivity (C_1_) retrogradely labeled from tibialis anterior (inset) and expressing Hb9::GFP (C_2_). **(D-G) **Cell body size distributions of all ChAT+ MNs (gray bars; 50 μm^2 ^bins, n = 3 animals; 481 ± 14.2 ChAT+ MNs analyzed per animal in six 70-μm thick ventral horn sections; error bars represent SEMs) with superimposed (white bars) distributions for the following subpopulations: all Gfrα1-TLZ+ MNs (D), Gfrα1-TLZ+ and Hb9::GFP- MNs (E), all Hb9::GFP+ MNs (F), MNs co-expressing Gfrα1-TLZ and Hb9::GFP (G). ChAT+ MNs were fit by two Gaussian curves of different widths representing small (D and E, red solid line) and large populations (D to G, blue solid lines). Two similar curves (dashed lines) fit Gfrα1-TLZ+ MNs. Most 'small' ChAT+ MNs are Gfrα1-TLZ+. Irrespective of Gfrα1-TLZ, Hb9::GFP+ neurons (solid red lines in F and G) display unimodal size distributions of averages and standard deviations resembling large ChAT+ MNs. Scale bars: (A) 100 μm; (B) 40 μm (also applies to (C)).

Immunohistochemical analysis of Gfrα1-TLZ heterozygous animals demonstrated that Gfrα1 is expressed most intensely in a subpopulation of relatively small neurons in lamina IX, which were independently identified as MNs by immunostaining for ChAT (Figure 1A) or retrograde labeling from hindlimb muscle (Figure 1B, C). To characterize the subpopulation of Gfrα1+ MNs, we compared the distribution of their cell sizes to that of all ChAT+ MNs. Gfrα1-TLZ staining was observed in the medial and lateral motor columns, but all quantitative analyses focused on the lateral motor column in the fourth and fifth lumbar spinal segments (L4-5) at P20. ChAT+ MN cell body sizes showed a bimodal distribution best fit by two Gaussian curves (correlation = 0.89; Figure 1D). The small size ChAT+ population had a mean average cross-sectional area of 331 ± 77 μm^2 ^( ± SD) while the large size population showed a wider distribution around a mean of 755 ± 220 μm^2^. The distribution of all Gfrα1-TLZ+ neurons was also best fit by a bimodal distribution (correlation = 0.9) with the same estimated parameters as the entire population of ChAT+ neurons (small population, 334 ± 82 μm^2^; large population, 754 ± 215 μm^2^). The percentage of ChAT+ neurons that were Gfrα1-TLZ positive was significantly different in the small versus large size groups. We used 485 μm^2 ^in area (μ + 2σ of the small population) as the cutoff point to distinguish the small and large populations. Using this criterion, small MNs represent 34 ± 1.4% ( ± SEM; n = 3 different animals) of all ChAT+ MNs, of which 91 ± 2% are strongly Gfrα1-TLZ positive (Figure 1E). In these L4-5 segments, approximately half (52 ± 6%) of the large ChAT+ MNs also express Gfrα1, but the intensity of Gfrα1-TLZ staining in many large MNs was relatively low (Figure 1C) and their numbers were variable in different pools. For example, in the case of specific dorso-lateral lumbar motor pools in caudal lumbar 5 and in the lumbar 6 segment, a large majority of large diameter MNs was weakly Gfrα1-TLZ+ (not shown).

We also observed that small ChAT+ MNs that displayed strong Gfrα1-TLZ immunoreactivity lacked Hb9::GFP expression, while MNs that were larger and weakly immunolabeled with Gfrα1-TLZ were frequently Hb9::GFP positive (Figure 1B, C). At P20, the cell body size distribution of Hb9::GFP+ neurons demonstrates that the transgene was selectively expressed in large, presumptive α-MNs (unimodal distribution around 817 ± 195 μm^2^) and represent approximately 66 ± 1% of all large MNs at this age (Figure 1F). Double-labeled Gfrα1-TLZ+/Hb9::GFP+ MNs were always distributed in the large population (Figure 1G). Thus, small, putative γ-type MNs are best identified by strong Gfrα1-TLZ expression and the absence of Hb9::GFP transgene expression. The cell body sizes of Gfrα1-TLZ+/Hb9::GFP- MNs are concentrated in the smaller size bins (Figure 1E), but their distribution shows a significant tail into large size bins and, as a result, is not well-fit by a Gaussian distribution. Gfrα1-TLZ+/Hb9::GFP- MNs comprise 89 ± 3% of small (<485 μm^2^) ChAT+ neurons and 24 ± 3% of large (>485 μm^2^) ChAT+ neurons at P20.

**Figure 2:**
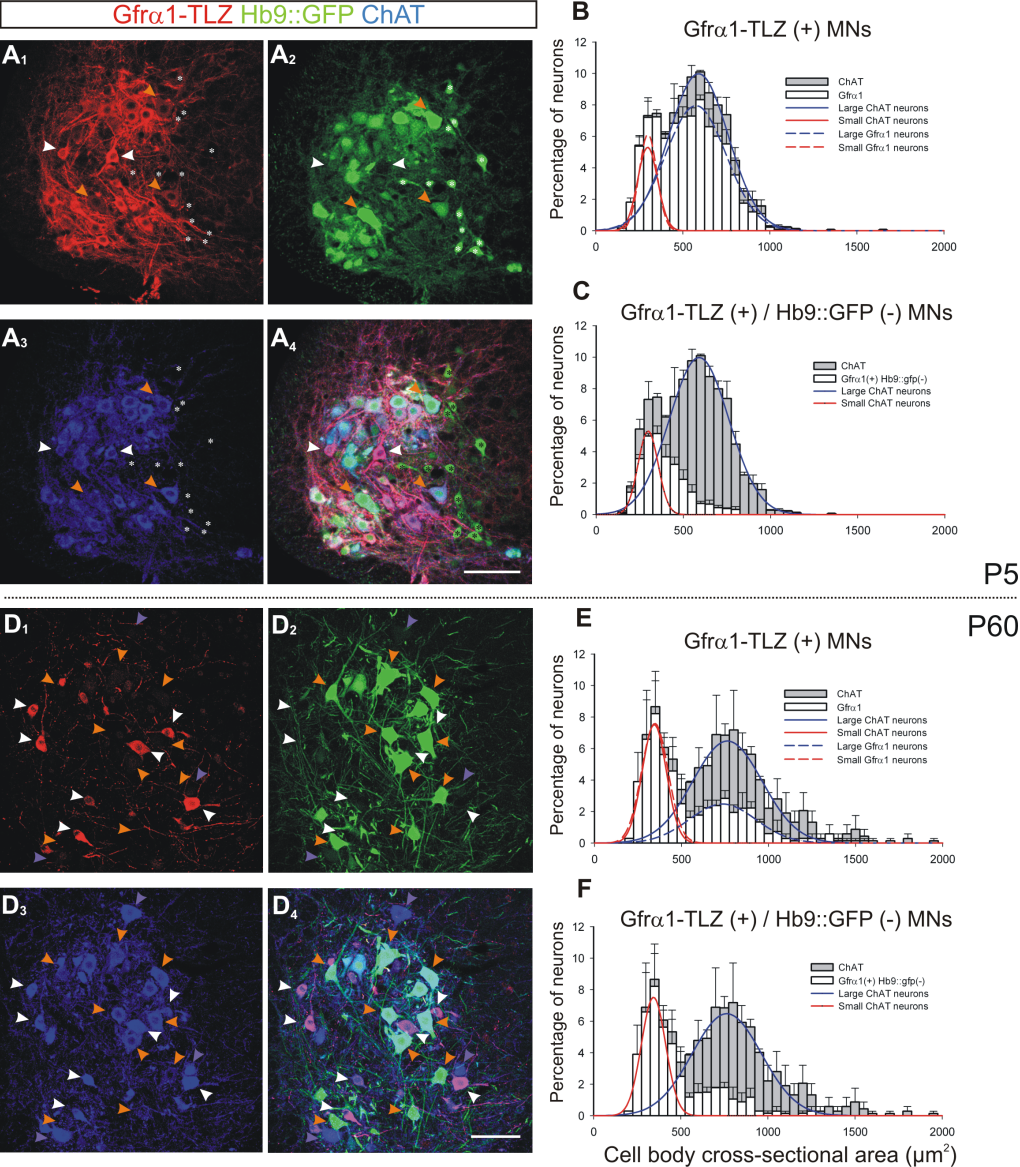
**Developmental downregulation of Gfrα1-TLZ and HB9::GFP expression**. **(A) **Single optical plane confocal image through lamina IX at P5 showing Gfrα1-TLZ expression (A_1_, Cy3, red), Hb9::GFP (A_2_, green), ChAT-immunoreactivity (A_3_, Cy5, blue) and merged images (A_4_). Most ChAT+ MNs express both markers at P5, but some express strong Gfrα1-TLZ and no Hb9::GFP (white arrowheads). A few large MNs express Gfrα1-TLZ weakly (orange arrowheads), while small Hb9::GFP interneurons (ChAT-, asterisks in A_-1-4_) do not express Gfrα1-TLZ. **(B) **Size distribution of P5 ChAT+ (gray bars) and Gfrα1-TLZ+ MNs (white bars); 83.6% of ChAT+ MNs express Gfrα1-TLZ. P5 ChAT+ MNs have small/medium sizes that can be fitted by two overlapping distributions (solid lines) suggesting initial differentiation of small (red line) and large (blue line) populations. Gfrα1+ MNs (dashed lines) are fitted by a similar bimodal distribution. **(C) **Size distribution of P5 Gfrα1-TLZ+ and Hb9::GFP- MNs (white bars). These cells represent 28.2% of all ChAT+ MNs (gray bars) and are concentrated in small size bins. **(D) **Similar image series as in A, but at P60 (images are at lower magnification and four optical planes were superimposed to adjust for neuropil spread with age). Gfrα1-TLZ+/Hb9::GFP- MNs (white arrowheads) are quite distinct at this age. Gfrα1-TLZ is largely absent from large MNs and Hb9::GFP+ MNs (orange arrowheads) and many large MNs also lack Hb9::GFP (blue arrowheads). **(E) **P60 size distributions (as in B). Only 54.1% of ChAT+ cells express Gfrα1-TLZ, with the strongest reduction in the large population. No significant downregulation of Gfrα1-TLZ expression occurs in small ChAT+ cells. **(F) **At P60, 43.9% of ChAT+ MNs are Gfrα1-TLZ+ and Hb9::GFP-. Their size distribution suggests many large Gfrα1-TLZ+ MNs have lost HB9::GFP. Error bars indicate SEM; 50 μm^2 ^bin size. At P5 average histograms from three animals, while at P60 two animals were averaged (six ventral horns analyzed per animal; 608 ± 7 and 337 ± 7 MNs analyzed per animal at P5 and P60 respectively). Scale bars: (A, B) 100 μm.

The expression of Gfrα1-TLZ and the expression of Hb9::GFP in MNs are independently regulated in postnatal development and become increasingly restricted with age to distinct but overlapping subpopulations (Figure 2; Tables 1 and 2). At P1 - even before there is a clear bimodal distribution of MNs - there is already a distinct population of lamina IX neurons at all segmental levels that express Gfrα1-TLZ strongly and lack Hb9::GFP, suggesting that the selective regulation of these genes in differentiating γ-MNs begins before birth. At P5 when a bimodal size distribution is first detected (Figure 2A-C), the majority of MNs express Gfrα1-TLZ (Figure 2B), but a distinct subpopulation of these Gfrα1-TLZ+ MNs is Hb9::GFP negative. These Gfrα1-TLZ+/Hb9::GFP- MNs are concentrated in the smaller cell size bins and represent 28% of all ChAT+ MNs (Figure 2C). By P20, the percentage of MNs that are Gfrα1-TLZ+ and Hb9::GFP- increases to 46% (Figure 1E) and then remains stable (44% at P60; Figure 2D-F). The percentage of MNs that are Gfrα1-TLZ+/Hb9::GFP- increases in the first postnatal weeks because of the progressive downregulation of Hb9::GFP expression in large MNs, including some weakly Gfrα1-TLZ+ MNs that are not γ-MNs (Figure 2D). At P60 the population of putative γ-MNs that are small, strongly Gfrα1-TLZ+ and Hb9::GFP- is well separated by size because of the relative postnatal growth of the larger Hb9::GFP+ α-MNs during postnatal development (Figure 2E-F).

**Table 1:** Percentage of ChAT immunoreactive motor neurons expressing each marker.

	P5	P20	P60
Gfrα1+	83.6 ± 2.3	65.0 ± 3.1	54.1 ± 0.9
Gfrα1+/Hb9::GFP-	28.2 ± 0.9	46.2 ± 0.6	43.9 ± 1.1
Hb9::GFP+	70.2 ± 0.6	45.5 ± 0.3	45.8 ± 0.5
Gfrα1+/Hb9::GFP+	55.3 ± 1.5	18.9 ± 3.3	10.2 ± 1.9

Units are percentages ± standard error of the mean. P, postnatal day.

**Table 2:** Statistical parameters of small and large populations fitted to all Lamina IX ChAT immunoreactive neurons of different ages.

	μ_1 _small	μ_2 _large	σ_1 _small	σ_2 _large
P5	297.5 ± 3.1*	590.0 ± 4.1*	57.5 ± 3.7	170.8 ± 4.5*
P20	334.2 ± 3.8	754.9 ± 9.1	77.6 ± 4.1	219.7 ± 10.9
P60	347.0 ± 3.2	763.8 ± 9.3	69.4 ± 4.6	199.8 ± 11.2

*Statistically significant difference (one-way ANOVA *P *< 0.05). Units are μm^2 ^± standard error of the mean. P, postnatal day.

**Figure 3:**
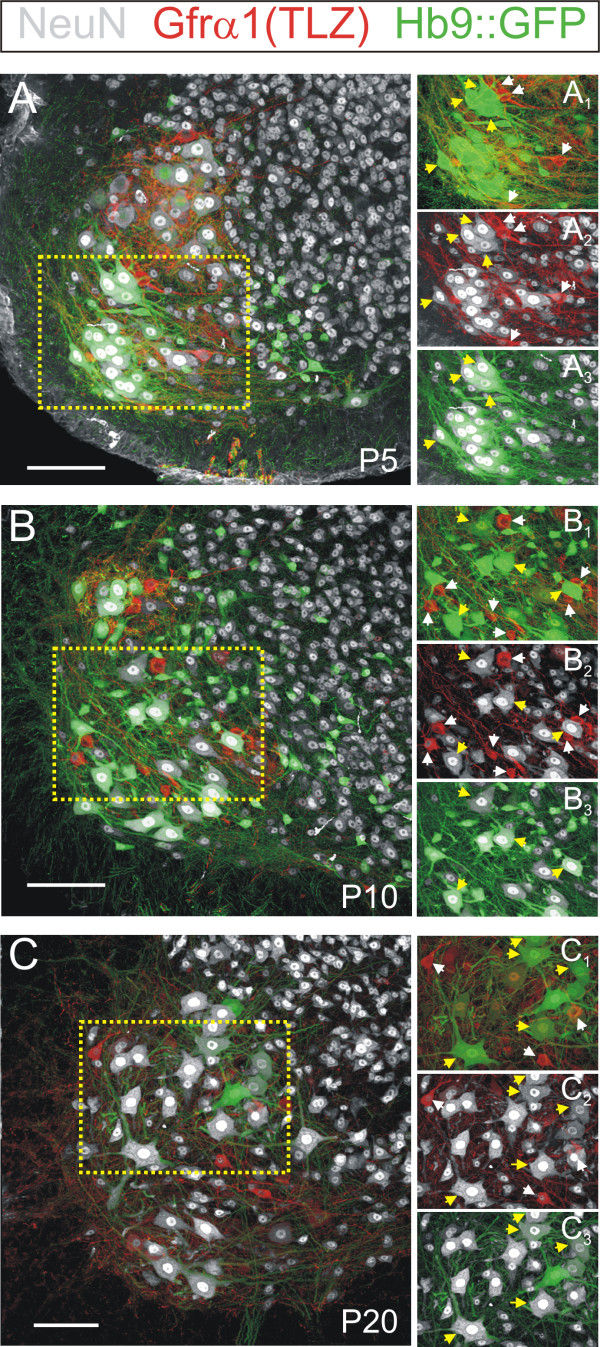
**NeuN is expressed at low levels or not at all in postnatal Gfrα1-TLZ+/Hb9::GFP- motor neurons**. **(A) **Confocal image of a P5 ventral horn showing Gfrα1-TLZ expression (Cy3, red), Hb9::GFP (green) and NeuN immunoreactive neurons (Cy5, white). All confocal planes through the 70 μm thick section and all fluorescent signals are superimposed. The lamina IX region enclosed by the dotted yellow box is shown in (A_1-3_) with Gfrα1-TLZ expression superimposed on Hb9::GFP (A_1_), Gfrα1-TLZ expression on NeuN (A_2_) and Hb9::GFP on NeuN (A_3_). **(B,C) **Similar image series for P10 (B,B_1-3_) and P20 (C,C_1-3_). At all ages, NeuN immunoreactivity is very low or not present at all in small MNs that are Gfrα1-TLZ+ and Hb9::GFP- (white arrows). In contrast, large Hb9::GFP+ MNs (yellow arrows) almost always express high levels of NeuN immunoreactivity. Scale bars: (A,B,C) 100 μm.

In the course of our MN cell counts, we also made the unexpected observation that small Gfrα1-TLZ+/Hb9::GFP- MNs generally lack immunoreactivity for the neuronal nuclear protein NeuN [31] (Figure 3). The large majority of Hb9::GFP+ MNs at P20 (96 ± 2% ( ± SEM); n = 4 animals) are NeuN positive, but only 31 ± 6% of Gfrα1+/Hb9::GFP- MNs contain NeuN immunoreactivity and this was always weak (Figure 3). Similar low percentages of weak NeuN immunoreactive (NeuN-IR) neurons were found in the Gfrα1-TLZ+/Hb9::GFP- population at P0 (19%; n = 2 animals), P5 (28%; n = 2) and P10 (14%; n = 3) (Table 3). Lack of NeuN in γ-MNs was also reported recently in an independent study [6].

**Table 3:** Percentage of neurons in each category that are NeuN positive.

	P0	P5	P10	P20
Hb9::GFP+	100	100	100	96 ± 1.7
Gfrα1+/Hb9::GFP-	18.7 ± 4.8	27.7 ± 2.6	13.6 ± 1.3	30.5 ± 5.5

Units are percentages ± standard error of the mean. P, postnatal day.

In conclusion, small MNs are generally characterized by strong Gfrα1-TLZ expression, low NeuN immunoreactivity and lack of Hb9::GFP. We hypothesize that these markers define new molecular criteria by which to identify γ-MNs at birth, well before other distinguishing features are expressed in mature α- and γ-MNs.

### Dendritic structure and synaptic inputs of Gfrα1+/Hb9::GFP- spinal neurons are typical of γ motor neurons

In addition to small size, mature γ-MNs are characterized by a distinct dendritic morphology [32-35], lack of primary afferent inputs [36-39] and absence of C-terminals contacting their somata [40-43].

**Figure 4:**
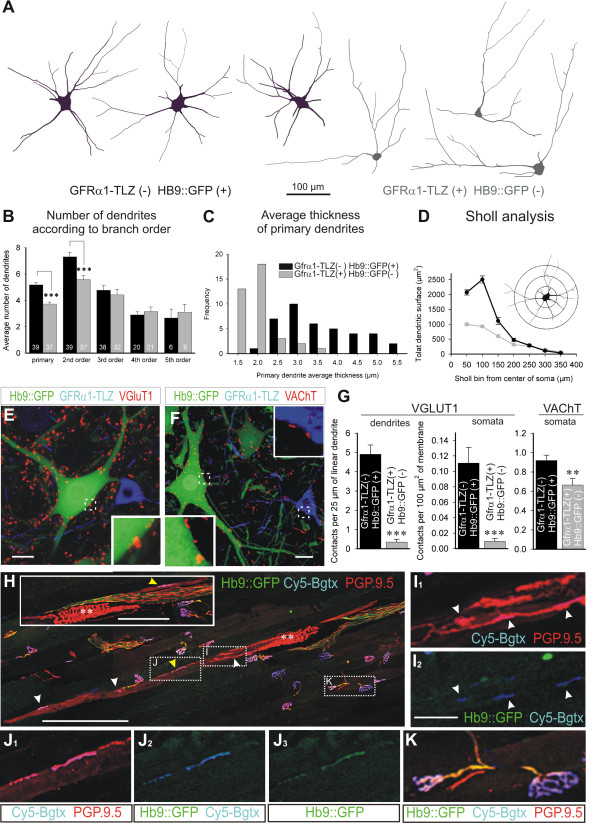
**Gfrα1-TLZ+/Hb9::GFP- motor neurons display structural and synaptic characteristics of gamma motor neurons**. **(A) **Neurolucida tracings of P20 large Gfrα1-TLZ-/Hb9::GFP+ (black) and small strongly Gfrα1-TLZ+/Hb9::GFP- MNs (gray). **(B-D) **Quantitative analyses of dendritic arbors. Gfrα1-TLZ-/Hb9::GFP+ MN primary dendrites are more numerous, more highly branched (B) and thicker (C), than those of Gfrα1+/Hb9::GFP- MNs. Sholl analysis (D) of Gfrα1-TLZ-/Hb9::GFP+ (black line) and Gfrα1-TLZ+/Hb9::GFP- (gray line) MNs also reveals differences in the distribution of membrane surface at different distances from soma that are characteristic of α- vs. γ-MNs. **(E) **VGluT1+ contacts (red) are present on P20 Hb9::GFP+ (green) MNs, but absent on Gfrα1-TLZ+ (blue)/Hb9::GFP- neurons. **(F) **VAChT+ contacts (red) are present on both Hb9::GFP+ (green) and Gfrα1-TLZ+ (blue)/Hb9::GFP- MNs (E and F, regions in white boxes are magnified in insets). **(G) **Quantification of dendritic and somatic VGluT1 and VAChT positive contacts on Hb9::GFP+ (black bars) and Gfrα1-TLZ+/Hb9::GFP- (gray bars) MNs (error bars indicate SEMs; triple and double asterisks indicate significance levels of *P *< 0.001 and *P *< 0.01 in t-test comparisons, respectively). **(H-K) **Tibialis anterior muscle in a Hb9::GFP mouse showing Cy5-bungarotoxin (Cy5-Bgtx, blue) labeled intra- and extrafusal neuromuscular junctions (NMJ) and PGP9.5 immunolabeled sensory and motor axons (red). Hb9::GFP+ motor axons are in green (H). Spindle afferent annulospiral endings (dual asterisks; also shown in the inset in a serial section) and intrafusal muscle fibers are labeled with PGP9.5. Extrafusal NMJs are innervated by PGP9.5+ and Hb9::GFP+ motor axons (K, high magnification of boxed area). Most motor end-plates on intrafusal fibers lacked GFP. Intrafusal Cy5-Bgtx NMJs (white arrowheads) are innervated by PGP9.5-IR axons that are HB9::GFP- (I, high magnification of boxed area). Yellow arrowheads indicate a few intrafusal NMJs innervated by PGP9.5+ and Hb9::GFP+ motor axons (example boxed and shown at higher magnification in J). Scale bars: (E,F) 20 μm; (H) 200 μm (100 μm in inset); (I,J) 25 μm.

To confirm the γ identity of Gfrα1-TLZ+/Hb9::GFP- MNs, we first compared their dendritic arborization with that of Hb9::GFP+ MNs (Figure 4A). At P20, Gfrα1-TLZ+/Hb9::GFP- neurons (n = 37) had significantly fewer primary and secondary dendrites (Figure 4B) than Gfrα1-TLZ-/Hb9::GFP+ MNs (n = 39) and these were always thinner at their origin (Figure 4C), a characteristic of γ-MNs [34]. In addition, Sholl analysis of Hb9::GFP+ MNs showed a rapid increase in dendritic surface in proximal dendritic segments followed by a slow decline as dendrites taper, a pattern that is characteristic of α-MNs [34,35] and distinct from that observed for Gfrα1-TLZ+/Hb9::GFP- MNs (Figure 4D). These results indicate that the dendritic arbor of Gfrα1-TLZ+/Hb9::GFP- MNs displays a γ-MN type of branching.

Second, using VGluT1 as a marker of primary afferent contacts [44-46] and VAChT to identify cholinergic terminals, we compared the synaptic inputs on these distinct MN populations. Gfrα1-TLZ+/Hb9::GFP- MNs had few VGluT1 contacts on their dendrites or somata in marked contrast to Gfrα1-TLZ-/Hb9::GFP+ MNs (Figure 4E, G; *P *< 0.001, *t*-tests). The somatic density of VAChT-IR contacts on Gfrα1-TLZ+/Hb9::GFP- MNs (Figure 4F) was only slightly less than that on Hb9::GFP+ MNs (difference in contact density was 27%, *P *< 0.01, *t*-test; Figure 4G); however, VAChT-IR contacts on Gfrα1-TLZ+/Hb9::GFP- MNs were much smaller in size. The average apposition lengths between VAChT-IR contacts and MNs was 2.45 ± 0.05 μm ( ± SEM) for Gfrα1-TLZ+/Hb9::GFP+ and 1.40 ± 0.06 μm for Gfrα1-TLZ+/Hb9::GFP- MNs (n = 291 and 137 VAChT-IR clusters counted on 10 MNs of each respective class; *P *< 0,001, *t*-test). These data describe a cholinergic input to Gfrα1-TLZ+/Hb9::GFP- MNs that is distinct from the large C-type cholinergic inputs previously identified by electron microscopy only on α-MNs. The dendritic morphology and synaptic input of the Gfrα1-TLZ+/Hb9::GFP- neurons are therefore consistent with their identification as γ-MNs.

### Gamma fusimotor axons are Hb9::GFP negative

In the rat, the incidence of γ and β efferents differ from muscle to muscle, but the large majority of motor inputs on intrafusal fibers in hindlimb muscles are γ motor axons [47]. Our findings in the Gfrα1-TLZ/Hb9::GFP mouse predict that these γ fusimotor endings should be TLZ+ and GFP-. Though we were unable to detect lacZ immunostaining in distal motor axons in the Gfrα1-TLZ animals, we could easily visualize GFP in muscle nerve by GFP immunostaining in Hb9::GFP+ animals and follow this marker to the neuromuscular junction. Neuromuscular junctions were visualized with a fluorescent α-bungarotoxin, and annulospiral primary sensory endings and motor axons were labeled with antibodies against PGP9.5. PGP9.5 is also weakly expressed in intrafusal muscle fibers, which made it possible to identify intrafusal motor endings in the juxtaequatorial and polar regions of the spindle (Figure 4H-K).

Using these markers, we analyzed extrafusal motor endings (n = 105) in the tibialis anterior muscle of a P30 double heterozygous Gfrα1-TLZ/Hb9::GFP mouse and found that all were GFP+. In contrast, 91% (30 of 33) of intrafusal neuromuscular junctions identified in 12 individual tibialis anterior muscle spindles were innervated by motor axons that were Hb9::GFP-. Since all motor axons that innervate extrafusal tibialis anterior muscle are Hb9::GFP+, so too must be any β-skeletofusimotor collateral. Hb9::GFP- fusimotor axons must therefore originate from γ-MNs, providing further evidence that postnatal γ-MNs do not express Hb9::GFP.

### Gamma motor neuron survival depends on target muscle spindles

**Figure 5:**
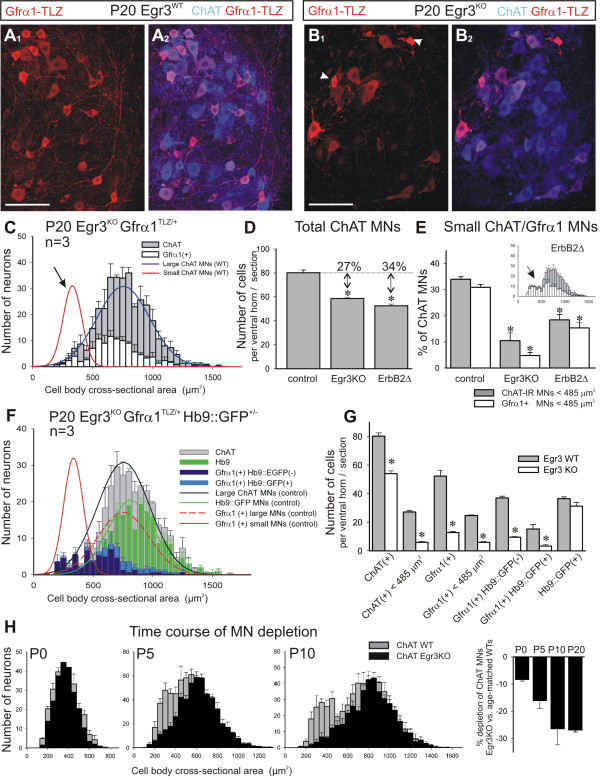
**Small diameter Gfrα1+ motor neurons are selectively lost in the muscle spindle mutant Egr3^KO ^and ErbB2^FLOX/-^myf5^Cre/+ ^animals**. **(A,B) **Lamina IX confocal images from a P20 Egr3 wild type (A) and Egr3^KO ^mutant (B). (A_1_, B_1_) Gfrα1-TLZ (red) and (A_2_,B_2_) superimposed with ChAT (blue). Small MNs intensely labeled with Gfrα1-TLZ are frequent in wild type but mostly absent in Egr3^KO ^mutants. Somatic shrinkage is apparent in the few small Gfrα1-TLZ MNs found in Egr3^KO ^mutants (arrowheads in B_1_). **(C) **Average size distribution of ChAT+ (gray bars) and Gfrα1-TLZ (white bars) MNs in P20 Egr3^KO ^animals (n = 3 animals; error bars indicate SEM). Superimposed curves represent the wild-type distributions of small and large MNs. ChAT+ and Gfrα1-TLZ+ populations in Egr3^KO ^mutants are both unimodal corresponding with large MNs. Small MNs are mostly absent (arrow). **(D) **Number of ChAT+ neurons sampled per ventral horn in 70-μm thick sections of P20 animals. Egr3^KO ^(n = 3 animals) and Erb2Δ animals (n = 5) show significant depletions compared to controls (asterisks indicate *P *< 0.001, one-way ANOVA; *P *< 0.01 *post-hoc *Tukey-tests). **(E) **Percentages of the total ChAT+ population represented by 'small' (<485 μm^2^) MNs labeled with ChAT (gray bars) or Gfrα1-TLZ (white bars) in Egr3^KO ^and Erb2Δ mutants. Both animals show a large depletion of small MNs compared to control (asterisks indicate *P *< 0.001, one-way ANOVA; *P *< 0.01 when compared to control using *post-hoc *Tukey-tests). In ErbΔ2 animals the reduction is not as pronounced as in Egr3^KO ^animals despite a larger depletion in total ChAT+ MNs (D). Inset shows the average size distribution histogram in ErbΔ2 animals (gray bars, ChAT+; white bars, Gfrα1+) suggesting partial depletion of both large and small MNs. **(F) **Average size distribution of MNs in Egr3^KO ^animals with different combinations of markers: ChAT+ (gray bars), Hb9::GFP+ (green bars), Gfrα1-TLZ+/Hb9::GFP- (dark blue bars) and Gfrα1-TLZ+/Hb9::GFP+ (light blue bars). Superimposed are the fitted distributions for different types of MNs in the wild-type. **(G) **Average number of MNs per ventral horn in each category. Asterisks denote significant differences (*P *< 0.001, t-test) between wild-type (gray bars) and Egr3^KO ^mutants (white bars). Significant differences were observed in all MNs and in small MNs (<485 μm^2^) labeled with either ChAT or Gfrα1-TLZ. Hb9::GFP+ MNs are not significantly depleted in Egr3^KO ^mutants, though Gfrα1-TLZ expression is downregulated in surviving large diameter MNs (F). **(H) **Time course of size differentiation and depletion of small MNs in Egr3^KO ^animals. Gray bars indicate size distribution in wild type (WT; n = 3 animals for each age) and black bars in age matched Egr3^KO ^animals (n = 2 at P0, n = 4 at P5 and P10). At P0, MN sizes are unimodal and there is a small depletion in ChAT+ neurons distributed in all size bins. At P5 there is initial differentiation of small vs. large MNs and in the Egr3^KO ^mutant there is a larger depletion of ChAT+ MNs concentrated in the small bins. At P10 the size distribution in the wild type resolves into two discrete peaks for the small and large population and in the Egr3^KO ^mutant the depleted neurons are clearly in the small size bins. Histogram at the right show the percentage depletions calculated in Egr3^KO ^mutants of different ages. Scale bars: (A_1_, B_1_) 100 μm.

In Egr3 mutant (Egr3^KO^) mice, early muscle spindle development is abnormal, and muscle spindles degenerate in the postnatal period [17,48]. This is accompanied by a loss of γ-axons in ventral roots and peripheral nerves. To determine whether there is a corresponding loss of γ-MN cell bodies, we generated Egr3^KO ^mice that were heterozygous for Gfrα1-TLZ^+/- ^(n = 3 animals) or double heterozygous for Gfrα1-TLZ^+/- ^and Hb9::GFP^+/- ^(n = 3) and analyzed the size distributions of L4-5 lumbar MNs expressing the different genetic markers. These were compared to Egr3 wild-type (Egr3^WT^) controls (n = 3) (Figure 5A-H). In Egr3^KO ^animals there was a selective loss of small ChAT+ neurons with strong Gfrα1-TLZ expression (Figures 5A-C). In equivalent sections of L4-5 spinal cord, the total number of ChAT+ MNs was reduced by 27% in Egr3^KO ^Gfrα1-TLZ^+/- ^animals compared to controls (Figure 5D; *P *< 0.001, *t*-test). This loss could be accounted for by 89% loss of small diameter, Gfrα1-TLZ+ MNs (Figure 5E, G). Moreover, in double heterozygous Egr3^KO ^animals the population of Gfrα1-TLZ+ and HB9::GFP- MNs was decreased by 74% (Figure 5G), and the few small Gfrα1-TLZ+ MNs surviving in these P20 animals showed significant somatic shrinkage, morphological evidence of degeneration (arrowheads in Figure 5B_1_). There was no significant decrease in the number of large diameter MNs in the Egr3^KO ^animals. Therefore, the proportion of small MNs (<485 μm^2^) that were Gfrα1-TLZ+ decreased from 31% ± 1 ( ± SEM) to 5 ± 1% of all ChAT+ MNs (Figure 5E). As a consequence, in the Egr3^KO ^mutants ChAT+ MNs comprise a single population best fit by a single Gaussian (correlation = 0.88; Figure 5C, F) with a mean average cross-sectional area of 754 ± 6 μm^2 ^( ± SEM), identical to the large MN population observed in wild-type controls.

In Egr3^KO ^mutants double-labeled with Gfrα1-TLZ and HB9::GFP (Figure 5F, G), there was no significant depletion of large ChAT+ MNs expressing Hb9::GFP (36.4 ± 1.3 GFP+ MNs per ventral horn in control compared to 31.3 ± 2.7 in Egr3^KO ^mutants; *P *= 0.154, *t*-tests). In contrast, the number of large MNs that were Gfrα1-TLZ+ and HB9::GFP+ declined by 78%. Since the total number of large ChAT+ and Hb9::GFP+ MNs is unchanged in Egr3^KO ^mutants, the decrease in the number of dual-labeled Gfrα1-TLZ+/and HB9::GFP+ MNs is best explained by downregulation of Gfrα1 expression in surviving MNs. This spindle dependence of Gfrα1 expression in large MNs together with our finding that all motor axons innervating extrafusal tibialis anterior muscle fibers are Hb9::GFP+ suggest that large Gfrα1-TLZ/Hb9::GFP MNs may be the source of β-skeletofusimotor axons.

To confirm the dependence of γ-MNs on muscle spindles, we analyzed a second mutant in which muscle spindle induction is inhibited by the conditional elimination of ErbB2 from embryonic muscle [22]. In these *ErbB2*^*NULL*/*FLOX*^*/myf5*^*CRE *^spindle mutants (ErbB2Δ in future text and figures), we also observed a marked 34% loss of ChAT+ MNs (*P *< 0.001, *t*-test), and a decrease of small (<485 μm^2^) Gfrα1-TLZ+ MNs to 15 ± 2% of the total. When expressed as a percentage of all ChAT+ MNs, there appears to be an intermediate loss of γ-MNs in the ErbB2Δ spindle mutant that is significantly different from that found in control and Egr3^KO ^animals (*P *< 0.001, one-way ANOVA). This difference can be explained by a significant decrease in the total number of large diameter MNs and the survival of some small MNs in the ErbB2Δ mutant (see inset in Figure 5E), suggesting broader effects of the ErbB2Δ mutation on MNs compared to Egr3 knockout. Nevertheless, together with data from the Egr3^KO ^animals, the loss of small Gfrα1+ MNs in the ErbB2Δ mutant confirms the target dependence of γ-MNs.

To determine the time course of γ-MN cell loss in the absence of normal spindle development, we analyzed the number and size distribution of ChAT+ MNs in neonatal (P0), P5 and P10 spinal cords from Egr3^KO ^mice. At birth, when size differences between ChAT+ MNs are not apparent (Figure 5H), there is a modest 8.0% loss of ChAT+ MNs in Egr3^KO^animals (n = 2 mutant animals compared to three controls) and this cell loss is distributed over all size bins. At P5, when a bimodal distribution of MN cell bodies is first evident, MN loss increases to 16% (*P *< 0.001, *t*-test; n = 4 mutants compared to 6 wild types at P5) mainly because of the selective loss of 65% (*P *< 0.001, *t*-test) of small diameter γ-MNs (<400 μm^2^; μ + 2σ of the estimated P5 small population) (Figure 5H). The loss of γ-MNs is more complete by P10, at which point 25% of all ChAT+ MNs are lost (n = 4 mutants compared to six wild-type animals at P10; *P *< 0.001, *t*-test), roughly equivalent to the loss observed at P20 (Figure 5H). These data suggest that γ-MNs begin to differentiate in Egr3^KO ^animals despite abnormal spindle development, but are progressively lost in the first postnatal week as spindles degenerate.

### Spindle-derived GDNF regulates the survival of γ motor neurons

**Figure 6:**
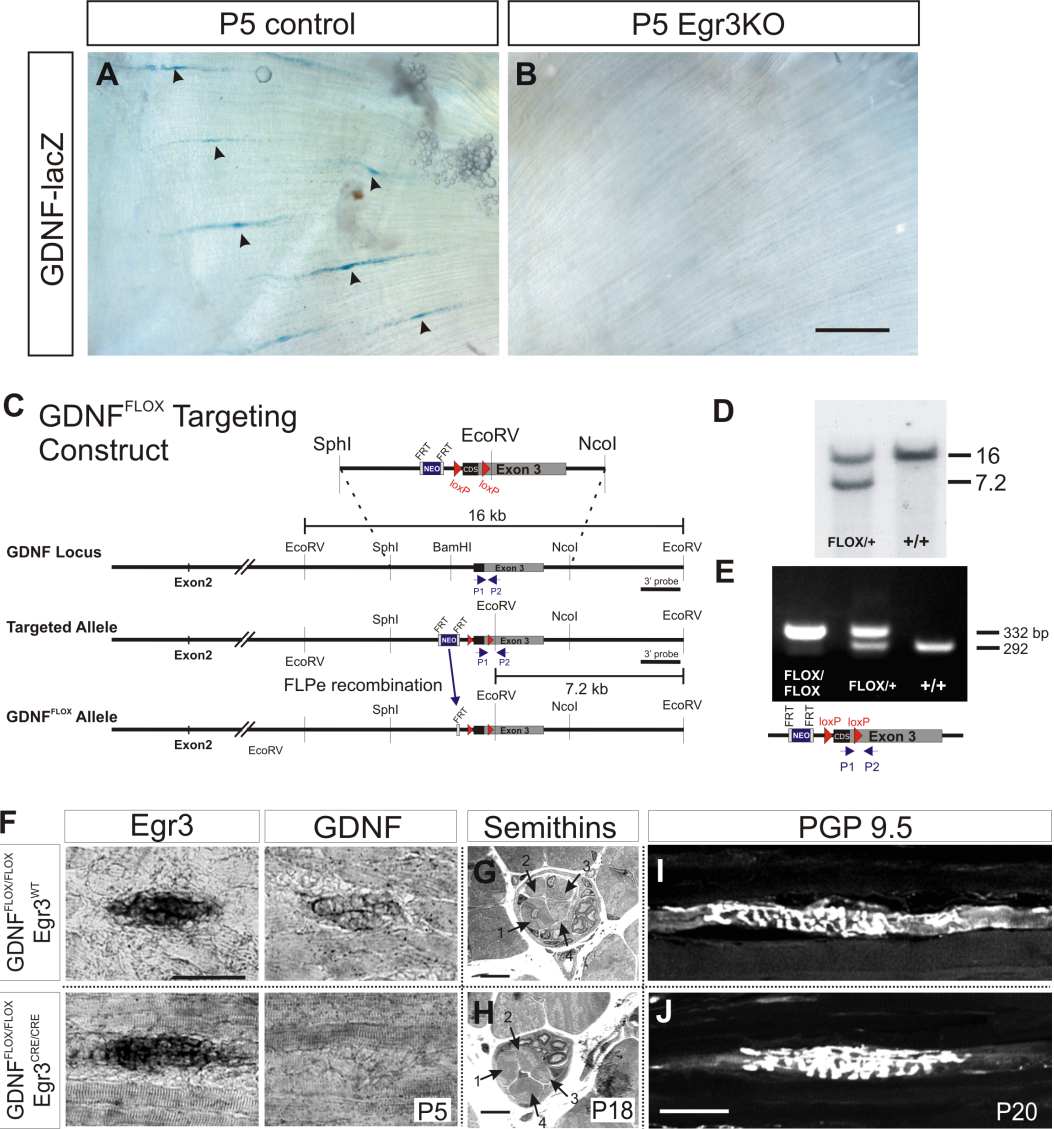
**Loss of muscle spindle-derived GDNF in the Egr3^KO ^and GDNF^FLOX^/Egr3^CRE ^conditional mutant mouse**. **(A,B) **GDNF (lacZ) is expressed in muscle spindles (black arrowheads) in P5 control gluteus maximus (*GDNF*^*lacZ*/-^*/Egr3*^+/-^) but absent in mutants (*GDNF*^*lacZ*/-^*/Egr3*^*KO*^). **(C) **Conditional gene targeting; *loxP *sites were introduced in the targeting construct around the *GDNF *gene coding sequence (CDS) before exon 3. An FRT-flanked neomycin-resistance (*Neo*) expression cassette was inserted upstream of the 5' *loxP *site and excised by crossing to ACTB-FLPe mice [14] to generate the *GDNF*^*FLOX *^allele. **(D) **Southern blot analysis of genomic DNA from mouse tails. Wild-type (+/+) and *GDNF*^*FLOX *^alleles are represented by 16 and 7.2 kb bands, respectively. **(E) ***GDNF*^*FLOX*/*FLOX *^and *GDNF*^*FLOX*/+ ^mice were identified by PCR using primers P1 and P2 (shown as arrowheads) that flank the 3' *loxP *inserted in the 3' untranslated region of the *GDNF *gene. **(F) ***In situ *hybridization of P5 *GDNF*^*FLOX*/*FLOX*^/*Egr3*^*CRE*/*CRE *^mutant and *GDNF*^*FLOX*/*FLOX*^*/Egr3*^*WT *^control hindlimb muscle spindles with probes for Egr3 and GDNF. Analysis was performed on 10 μm-thick contiguous cryosections to demonstrate co-expression of Egr3 and GDNF in control and lack of GDNF expression in mutant muscle spindles after Cre recombination. **(G,H) **Semithin (1 μm) sections showing that control (G) and mutant spindles (H) have the same number of intrafusal muscle fibers (indicated by numbered arrows). **(I,J) **PGP9.5-immunoreactive annulospiral endings are similar in P20 *GDNF*^*FLOX*/*FLOX *^(no Cre) control (G) and in *GDNF*^*FLOX*/*FLOX*^/*Egr3*^*CRE*/*CRE *^animals (H). Scale bars: (B) 200 μm; (F), 50 μm; (H) 10 μm; (J) 50 μm.

To test whether the loss of spindle-derived GDNF could account for the selective loss of γ-MNs we observed in the muscle spindle mutants, we first analyzed GDNF expression in muscle spindles in Egr3^KO ^and ErbB2Δ animals using a targeted allele of *GDNF *(*GDNF*^*LacZ*^) in which β-galactosidase (lacZ) expression replaces GDNF [21]. *GDNF*^*LacZ *^was crossed into the Egr3^KO ^background, and hindlimb muscles analyzed at P5 for GDNF (lacZ) expression. Histochemical staining for β-galactosidase activity revealed an absence of GDNF in postnatal Egr3^KO ^mutant spindles (Figure 6A, B), demonstrating that GDNF expression is dependent on Egr3 function in the program of muscle spindle differentiation. Similar results were found in the rudimentary spindles of ErbB2Δ mutants at P5. These findings provide indirect evidence that spindle-derived GDNF is required for γ-MN survival.

To address the question of whether spindle-derived GDNF is required for γ-MN survival, we generated a conditional allele of *GDNF *(*GDNF*^*FLOX*^; Figure 6C-E) and crossed the GDNF^*FLOX *^mouse to the Egr3-IRES-Cre (Egr3^CRE^) line [22] to selectively eliminate GDNF expression from muscle spindles. *GDNF*^*FLOX*/*FLOX*^*/Egr3*^*CRE*/*CRE *^mutant animals had no apparent phenotype at birth and are viable and mature into adulthood.

To determine whether Egr3^CRE ^could effectively eliminate GDNF expression in the *GDNF*^*FLOX*/*FLOX*^*/Egr3*^*CRE*/*CRE *^mice, we used *in situ *hybridization analysis to examine the expression of GDNF and the transcription factor Egr3, expressed in nascent intrafusal fibers upon muscle spindle induction at E15.5 [24]. In *GDNF*^*FLOX*/*FLOX *^(no CRE) controls, *in situ *hybridization analysis performed on contiguous sections of P5 hindlimb muscle revealed the co-expression of Egr3 and GDNF in muscle spindles (Figure 6F, top panels). In contrast, spindles identified by the expression of Egr3 in the *GDNF*^*FLOX*/*FLOX*^*/Egr3*^*CRE*/*CRE *^mutant did not co-express GDNF (Figure 6F, bottom panels).

Despite the loss of spindle-derived GDNF, the overall structure of muscle spindles was normal in these animals. Analysis of serial semithin sections through polar and equatorial regions of individual muscle spindles (n = 10 control, n = 6 mutant) revealed that mutant muscle spindles each contain four intrafusal fibers (Figure 6G, H), two chain and two bag cells, identical to controls. Immunostaining for PGP9.5 also showed that intrafusal fibers in mutant spindles are innervated by annulospiral afferent terminals similar in overall morphology to controls (Figure 6I, J). Therefore, at the light microscopy level, there were no obvious alterations in intrafusal or sensory afferent fiber composition and morphology.

**Figure 7:**
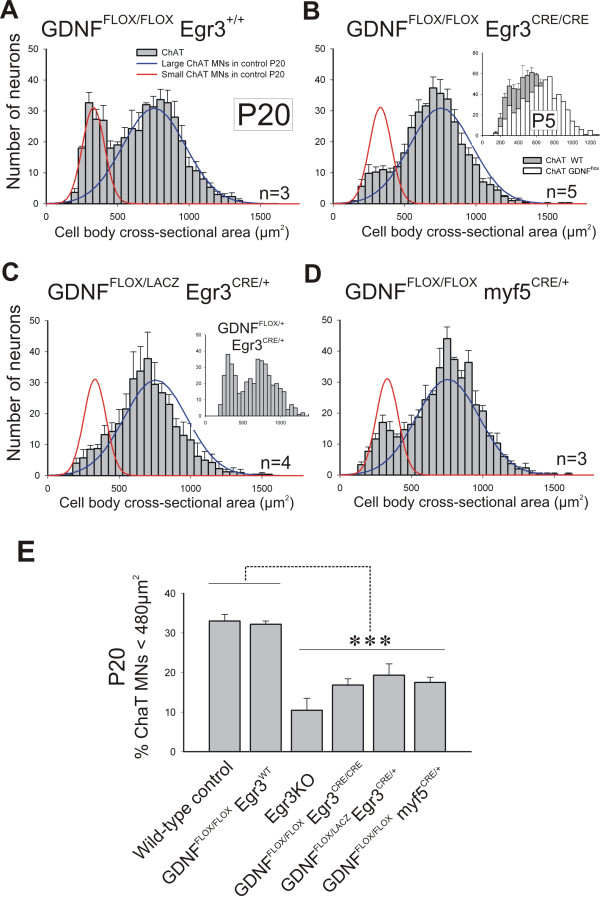
**Genetic elimination of muscle spindle-derived GDNF results in selective loss of gamma motor neurons**. **(A) **Size distributions of ChAT+ MNs in *GDNF*^*FLOX*/*FLOX*^*/Egr3*^*WT *^(no Cre) controls at P20 are comparable to wild types (lines). **(B) **ChAT+ MNs losses in the absence of spindle-derived GDNF (*GDNF*^*FLOX*/*FLOX*^*/Egr3*^*CRE*/*CRE *^mutants). Small ChAT+ MNs represent 32 ± 1% ( ± SEM) of all MNs in *GDNF*^*FLOX*/*FLOX *^*/Egr3*^+/+ ^and 16.8 ± 1.1% in *GDNF*^*FLOX*/*FLOX*^*/Egr3*^*CRE*/*CRE *^animals. Inset shows a depletion at P5 comparable to Egr3^KO ^animals (see Figure 5F). **(C) **Similar loses in compound heterozygotes with one conditional and one null GDNF allele and a single copy of *Egr3*^*CRE *^(*GDNF*^*FLOX*/*LACZ*^*/Egr3*^*CRE*/+^). Inset shows a normal size distribution in one animal carrying one wild type and one floxed *GDNF *allele and a single *Egr3*^*CRE *^copy. **(D) **GDNF elimination from all muscle precursors using *myf5*^*CRE*/+ ^results in similar losses of small ChAT+ MNs. Large MN numbers are unaffected in conditional mutants by targeted removal of GDNF from spindles. **(E) **Comparison of the percentage of small MNs (<480 μm^2^) in different genotypes. No differences were detected between wild-type and homozygous *GDNF*^*FLOX *^(no Cre) controls. Egr3^KO ^mutants and several conditional/floxed GDNF mutants crossed to *Egr3*^*CRE *^or *myf5*^*CRE *^showed significant depletions compared to wild-types and *GDNF*^*FLOX *^(no Cre) controls (asterisks indicate *P *< 0.001 one-way ANOVA followed by *P *< 0.01 *post-hoc *Tukey comparisons). Depletion of small MNs in Egr3^KO ^animals were more pronounced than in other genotypes, but differences were not statistically significant. N's, number of animals analyzed in each genotype.

Size histograms of ChAT+ MNs revealed a normal bimodal distribution in P20 *GDNF*^*FLOX*/*FLOX *^controls (n = 3) and no significant difference in the calculated size average or SD of the small and large populations in these animals compared to the fitted distributions of age-matched wild-type animals analyzed previously (Figure 7A). In contrast, *GDNF*^*FLOX*/*FLOX*^*/Egr3*^*CRE*/*CRE *^animals (n = 5) showed a significant (approximately 50%; *P *< 0.001, *t*-test) decrease in the number of small (<485 μm^2^) ChAT+ MNs (Figure 7B, E). This reduction was smaller than, but statistically not different from, that observed in Egr3^KO ^mutants (Figure 7E). Moreover, γ-MN depletion occurred with a similar time course in *GDNF*^*FLOX*/*FLOX*^*/Egr3*^*CRE*/*CRE *^animals (see inset in Figure 7B) compared to Egr3^KO ^animals (Figure 5H). As a result, ChAT+ MNs in the *GDNF*^*FLOX*/*FLOX*^*/Egr3*^*CRE*/*CRE *^mutant comprise a single population well-fit by a single Gaussian (correlation = 0.86) with a mean average cross-sectional area of 708 μm^2 ^± 200 ( ± SD), which is similar to that estimated for the large MN population in *GDNF*^*FLOX*/*FLOX *^controls (758 ± 201 μm^2^). Similarly, selective loss of γ-MNs was also observed in mutants carrying a conditional (*GDNF*^*FLOX*^) and null (*GDNF*^*LacZ*^) allele of *GDNF *and a single copy of *Egr3*^*CRE *^(Figure 7C, E).

Finally, to demonstrate that the selective loss of fusimotor neurons was due to the targeted elimination of GDNF from intrafusal muscle and not Schwann cells where GDNF [49] and *Egr3*^*CRE *^[22] are also expressed, we repeated this experiment with the muscle-specific Cre line, *myf5*^*CRE *^[20], and again consistently found a selective loss of small diameter (<485 μm^2^) MNs (n = 3; Figure 7D) that was not significantly different from that observed with Egr3^CRE ^(Figure 7E).

## Discussion

The study of muscle spindle function in motor control dates back to the first description of small diameter 'γ ' motor fibers in 1930 [50], but important questions remain about the development and significance of a system capable of controlling muscle spindle sensitivity independent of muscle contraction. In molecular terms, little is known about the mechanisms that control the differentiation of γ-MNs and determine their unique identity. To begin to address these questions, we have characterized several molecular genetic markers of γ-MN identity - high expression of Gfrα1 and low expression of the Hb9::GFP transgene and NeuN - and demonstrate the selective dependence of fusimotor neuron survival on target muscle spindle-derived GDNF in the early postnatal period. With these markers we also show that MNs from which β-skeletofusimotor axons originate survive in the absence of muscle spindles but downregulate Gfrα1 expression as they become pure α-MNs, innervating only extrafusal muscle. Finally, we take advantage of the selective trophic dependence of γ-MNs to establish a mouse model with which we can begin to explore the role of γ fusimotor activity in motor behaviors.

### GDNF dependence of γ motor neuron survival

MN identity is established during development by segregation into columns, divisions and ultimately pools of neurons that innervate individual target muscles (for a review, see [51]). Even within a motor pool, MNs can be further divided into those that innervate extrafusal muscle, and those that only innervate the intrafusal fibers of the muscle spindle, the γ-MNs. Though often not distinguished, γ-MNs differ from α-MNs in several fundamental ways, such as size, dendrite morphology, target choice, electrophysiological properties and synaptic organization. Yet we know little about the mechanisms that control γ-MN differentiation. One recent study demonstrates that the acquisition of GDNF dependence is a very early step in the functional differentiation of fusimotor neurons [12]. When GDNF signaling is disrupted in all MN precursors, differentiating γ-MNs are selectively lost during the period of programmed cell death by a mechanism that is likely mediated by the anti-apoptotic protein bcl-2 [52]. This occurs before the induction of muscle spindles at E15.5, which indicates that embryonic neurons committed to a γ-MN fate depend on early source(s) of GDNF other than the muscle spindle. In the study by Gould *et al*. [12] it was also concluded that the dependence of γ-MNs on GDNF signaling does not extend beyond P5 because no MNs are lost when the GDNF co-receptor gene *Ret *is conditionally deleted between P5 and 10. However, our data demonstrate that muscle spindles are a critical source of GDNF required for the survival of γ-MNs in this same postnatal period. Our conclusion is based on genetic studies in which we selectively deleted GDNF from muscle spindles using a novel conditional (floxed) *GDNF *allele and both muscle- and spindle-specific Cre drivers (Figure 7). The results were highly consistent in all 12 P20 animals in which GDNF was deleted from muscle spindles. The explanation for the contrasting conclusions with Gould and colleagues' study is unclear, but may relate to the timing and efficiency of Cre-mediated genetic deletion. That is, functional deletion of *Ret *by the inducible β-actin-Cre used by Gould and colleagues may not occur in γ-MNs or occur at a time when spindle-derived GDNF is no longer required for survival. Alternatively, spindle-derived GDNF may be required well in advance of the observed postnatal γ-MN degeneration, though we find no precedent for such a delayed response to the removal of trophic support.

### Molecular development of γ motor neurons and the role of spindle-derived factors

In the absence of GDNF signaling, γ-MNs degenerate selectively and no loss of α-MNs is observed [12]. Yet, Gfrα1 is expressed in many postnatal large diameter MNs, albeit at lower levels, which may reflect the differential dependence of some α-MNs on GDNF signaling [12,53] or the role of GDNF in other aspects of MN development - for example, cell body position, dendrite patterning and connectivity, motor axon projection and target innervation [25,53-55].

An additional role for GDNF signaling in MNs is suggested by our analysis of large Gfrα1+ MNs in the Egr3^KO ^mutant, which supports a role for GDNF in the specification of β-skeletofusimotor neurons. In Egr3^KO ^animals, all large MNs survive, but downregulate Gfrα1, perhaps because of the loss of spindle-derived GDNF. This spindle-dependence of Gfrα1 expression argues that large Gfrα1+ MNs interact directly with muscle spindles and therefore must represent those MNs that send a β-skeletofusimotor collateral to intrafusal muscle fibers. Our analysis also shows that β efferents are Hb9::GFP+, and together these findings argue that the subpopulation of MNs that co-express Gfrα1-TLZ and Hb9::GFP are β-skeletofusimotor neurons that innervate both intra- and extrafusal muscle. The variable numbers of mature Gfrα1-TLZ+/Hb9::GFP+ MNs we observed in different pools may then reflect differences in the amount of β-innervation in different muscles. Spindle-derived factors may maintain β-skeletofusimotor collaterals and regulate aspects of γ-MN differentiation - for example, strong Gfrα1 expression, Err3 expression or downregulation of NeuN - but the degeneration of muscle spindles and the selective loss of γ-MNs in the Egr3^KO ^mutant precludes this analysis.

The differential expression of the *Hb9::GFP *transgene in large diameter MNs is regulated independently of Gfrα1 expression and is not influenced by spindle-derived factors. Moreover, *Hb9::GFP *transgene expression does not faithfully reflect the expression of the endogenous *Hb9 *gene, which analysis of the Hb9-NLS-LacZ knock-in mice demonstrates is expressed in both α- and γ-MNs [6]. Extensive ectopic expression of the *Hb9::GFP *transgene has been reported in non-Hb9 interneurons in lumbar segments [29,30], suggesting that regulatory elements of the *Hb9 *gene are missing in the transgene that could also account for its consistent, selective downregulation in postnatal γ-MNs.

Our data provide further evidence that reciprocal interactions between the muscle spindle and the sensory and MNs that innervate it are critical to establish and maintain the circuits that provide proprioceptive sensory feedback during motor behaviors. Primary afferents induce muscle spindles through a mechanism dependent on neuronal Neuregulin 1 (Nrg1) [24]. In response to Nrg1 signaling, early myocytes differentiate into intrafusal muscle by a program that is dependent in part on the activity of the transcription factor Egr3 [17]. In the absence of Egr3, muscle spindles fail to express Neurotrophin 3 (NT-3) [56], which muscle spindle afferents require to maintain functional monosynaptic connections with MNs in the postnatal period [22,56]. In a similar way, muscle spindles also serve as a late source of GDNF, which is required for the survival of γ-MNs and may regulate some properties of β-skeletofusimotor axons as well.

### Transcriptional profile of γ motor neurons

Some GDNF actions on MN development are mediated by induction of the ETS transcription factor Pea3 [54,55,57]. In the mouse embryo, Pea3 is localized only to certain motor pools [58] in a pattern that is not consistent with the widespread distribution of fusimotor neurons in most motor pools; this is also the case in the postnatal spinal cord (NAS, unpublished observation). In its role in fusimotor neuron development, GDNF apparently functions through alternative transcriptional pathways independent of Pea3. The recent report that Err3 is restricted to postnatal γ-MNs [6] suggests a role for this transcription factor in γ-MN differentiation. But like Gfrα1 and Hb9::GFP, the differential regulation of Err3 in γ-MNs occurs in the first weeks after birth so it does not appear to function in the earliest specification steps.

The transcriptional profile of γ-MNs may also be reflected in the selective downregulation of NeuN, a predominantly nuclear protein that is able to bind DNA and is expressed exclusively in postmitotic neurons [31]. The recent report that NeuN is not expressed in postnatal γ-MNs [6] conflicts somewhat with our finding of low NeuN levels relative to α-MNs. This may be a question of sensitivity of detection, but nevertheless both studies are in agreement in that NeuN immunoreactivity is significantly weaker in γ-MNs compared to α-MNs. The mechanisms regulating the expression of NeuN in γ-MNs are not understood, but other specific neuronal populations, including Purkinje cells, mitral cells and most retinal cells, in the inner granular layer also lack NeuN immunoreactivity [31]. NeuN immunodetection is also reduced or abolished after neuronal injury [59] and significantly decreased in MNs after axotomy [60]. Though its exact nature and function are unknown, NeuN is found in areas of low chromatin density [61] and may directly or indirectly relate to the state of chromatin, which controls distinct patterns of gene expression involved in neural development [62]. It is therefore tempting to suggest that NeuN-related epigenetic mechanisms are part of a program that regulates γ-MN differentiation and several molecular genetic aspects of fusimotor identity.

### Functional implications

In contrast to Egr3^KO ^animals [17] and mutants lacking spindle-derived Neurotrophin 3 [22], mice without spindle-derived GDNF have no apparent defects during normal, unchallenged locomotion. This is consistent with our demonstration that muscle spindles and their afferent terminals are structurally normal in GDNF^FLOX/FLOX^/Egr3^CRE/CRE ^mice. The absence of an obvious motor phenotype in animals in which γ-MNs are significantly depleted could reflect residual γ-fusimotor activity or functional compensation by β-skeletofusimotor inputs to muscle spindles. However, the lack of an overt phenotype during normal locomotion on level ground, as observed, for example, during slow speed treadmill locomotion, may reflect low level recruitment of γ-control during this type of locomotion. Further study of these mutants using locomotor and other behavioral tasks that require dynamic regulation of muscle spindle sensitivity [5] are needed to demonstrate the specific role of the γ-fusimotor system in motor control.

## Conclusion

At birth, γ-MNs express high levels of Gfrα1 and low levels of NeuN and the *Hb9::GFP *transgene. Together, these define a unique molecular criterion for γ-MN identity. The strong expression of Gfrα1 in postnatal γ-MNs correlates with our finding that γ-fusimotor neurons depend selectively on muscle spindle-derived GDNF for their survival. In demonstrating this trophic dependence in mice, we created a novel animal model in which γ-MNs are selectively lost. Unlike other animals with muscle spindle or proprioceptor defects, this mutant preserves muscle spindle structure, sensory afferent innervation, and functional sensorimotor connectivity with no α-MN loss and so provides a genetic model to study the specific role of γ-MNs in motor control.

## Abbreviations

ChAT: choline acetyl transferase; E: embryonic day; Gfrα1: GDNF receptor; GDNF: glial cell line-derived neurotrophic factor; GFP: green fluorescent protein; IR: immunoreactive; MN: motor neuron; NeuN: neuronal nuclear protein; P: postnatal day; PBS: phosphate buffered saline; PGP9.5: protein gene product 9.5; SD: standard deviation; SEM: standard error of the mean; VAChT: vesicular acetylcholine transporter; VGluT1: vesicular glutamate transporter 1.

## Competing interests

The authors declare that they have no competing interests.

## Authors' contributions

NAS and FJA conceived the study, designed the experiments, analyzed the data and wrote the manuscript. NAS, MNB and CAS performed the experiments. CAS performed all neurolucida analyses of cell size and morphology. JP and NAS generated the *GDNF*^*FLOX *^mouse. All authors read and approved the final manuscript.
